# Open Reading Frame 4 Is Not Essential in the Replication and Infection of Genotype 1 Hepatitis E Virus

**DOI:** 10.3390/v15030784

**Published:** 2023-03-18

**Authors:** Huimin Bai, Yasushi Ami, Yuriko Suzaki, Yen Hai Doan, Masamichi Muramatsu, Tian-Cheng Li

**Affiliations:** 1 Department of Basic Medicine and Forensic Medicine, Baotou Medical College, Baotou 014060, China; 2 Division of Experimental Animals Research, National Institute of Infectious Diseases, Tokyo 208-0011, Japan; 3 Center for Emergency Preparedness and Response, National Institute of Infectious Diseases, Tokyo 208-0011, Japan; 4 Department of Virology II, National Institute of Infectious Diseases, Tokyo 208-0011, Japan

**Keywords:** hepatitis E virus, genotype 1, HEV-1 mutant, ORF4 mutant, reverse genetic system, PLC/PRF/5 cells, Mongolian gerbil

## Abstract

Genotype 1 hepatitis E virus (HEV-1), unlike other genotypes of HEV, has a unique small open reading frame known as ORF4 whose function is not yet known. ORF4 is located in an out-framed manner in the middle of ORF1, which encodes putative 90 to 158 amino acids depending on the strains. To explore the role of ORF4 in HEV-1 replication and infection, we cloned the complete genome of wild-type HEV-1 downstream of a T7 RNA polymerase promoter, and the following ORF4 mutant constructs were prepared: the first construct had TTG instead of the initiation codon ATG (A2836T), introducing an M→L mutation in ORF4 and a D→V mutation in ORF1. The second construct had ACG instead of the ATG codon (T2837C), introducing an M→T mutation in ORF4. The third construct had ACG instead of the second in-frame ATG codon (T2885C), introducing an M→T mutation in ORF4. The fourth construct contained two mutations (T2837C and T2885C) accompanying two M→T mutations in ORF4. For the latter three constructs, the accompanied mutations introduced in ORF1 were all synonymous changes. The capped entire genomic RNAs were generated by in vitro transcription and used to transfect PLC/PRF/5 cells. Three mRNAs containing synonymous mutations in ORF1, i.e., T2837C^RNA^, T2885C^RNA^, and T2837C/T2885C^RNA^, replicated normally in PLC/PRF/5 cells and generated infectious viruses that successfully infected Mongolian gerbils as the wild-type HEV-1 did. In contrast, the mutant RNA, i.e., A2836T^RNA^, accompanying an amino acid change (D937V) in ORF1 generated infectious viruses upon transfection, but they replicated slower than the wild-type HEV-1 and failed to infect Mongolian gerbils. No putative viral protein(s) derived from ORF4 were detected in the wild-type HEV-1- as well as the mutant virus-infected PLC/PRF/5 cells by Western blot analysis using a high-titer anti-HEV-1 IgG antibody. These results demonstrated that the ORF4-defective HEV-1s had the ability to replicate in the cultured cells, and that these defective viruses had the ability to infect Mongolian gerbils unless the overlapping ORF1 was accompanied by non-synonymous mutation(s), confirming that ORF4 is not essential in the replication and infection of HEV-1.

## 1. Introduction

Hepatitis E virus (HEV) is a quasi-enveloped (blood or tissue culture) or non-enveloped (feces) virus [1,2]. HEV contains a positive-sense single-strand RNA as the genome and has been classified in the family *Hepeviridae* [3]. Novel HEV strains have been identified in many animal species in recent years, and the genetic diversity within HEVs has been disclosed [4,5,6,7,8,9]. The current taxonomy shows that the family *Hepeviridae* includes two subfamilies: *Orthohepevirinae* and *Parahepevirinae*. The subfamily *Orthohepevirinae* includes at least four genera: *Paslahepevirus*, *Rocahepevirus*, *Chirohepevirus*, and *Avihepevirus* (ictv.global/taxonomy) [1]. The *Paslahepevirus* is classified into two species, *alci* and *balayani*, and the species *balayani* includes at least eight viral genotypes, HEV-1 to HEV-8, that infect humans and domestic and wild mammals. *Rocahepeviruses* infect rats and ferrets, and rat HEV is known to be transmitted to humans [10]. *Chirohepeviruses* are detected in bats, and *Avihepeviruses* are detected in birds [11,12]. No evidence has indicated that HEV is transmitted from bats to humans or from birds to humans.

Human HEV, a major cause of acute hepatitis, is transmitted by the consumption of contaminated water, undercooked or raw meat, and through blood transfusion or organ transplantation [13]. Although human HEV infection typically results in an acute and self-limited hepatitis, immunocompromised and transplant patients are vulnerable to prolonged infections and tend to develop chronic hepatitis [14].

**Figure 1:**
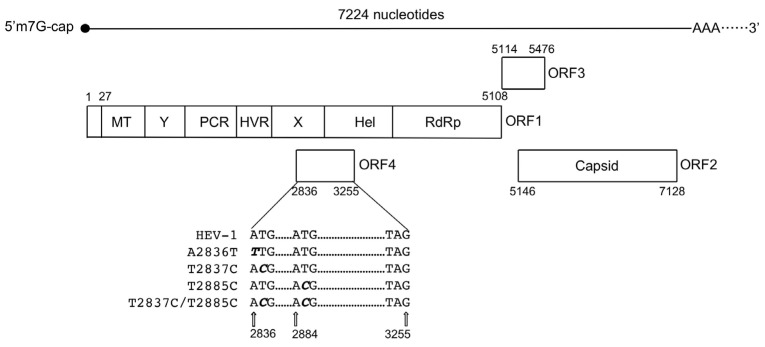
HEV-1 (LC061267) genome organization and the position of the nucleotide mutations. ORF1 to ORF4 and the putative functional domains observed in ORF1 are depicted. Hel: helicase, HVR: hypervariable region, MT: methyltransferase, PCP: papain-like cysteine protease, RdRp: RNA-dependent RNA polymerase, X: X-domain, and Y: Y-domain. The numbers indicate the nucleotide position from the 5′-end. Mutated nucleotides are shown by *bold* and *italics*.

The HEV genome is 6.4–7.2 kb in length and contains three major open reading frames (ORFs) flanked by short 5′- and 3′-terminal non-coding regions [1]. The 5′-end is m7 G-capped, and the 3′-end is polyadenylated (Figure 1). ORF1 encodes a non-structural polyprotein containing domains that are consistent with a methyltransferase (MT), a Y-domain, a papain-like cysteine protease (PCP), a hypervariable region (HVR), an X-domain, a helicase (Hel), and an RNA-dependent RNA polymerase (RdRp) [15]. ORF2 encodes the viral capsid protein. ORF3 encodes a phosphoprotein that interacts with the cellular cytoskeleton and is associated with virion release [16]. ORF1, ORF2, and ORF3 are commonly conserved in all HEV-related viruses. A putative ORF4 has been identified in rat HEV [5,17], ferret HEV [18], and HEV-1 [19,20], but the ORF4′s functions is unknown. The ORF4 of rat HEV and ferret HEV overlaps with the carboxyl terminal region of ORF1, and the ORF4 of HEV-1 overlaps with the X-domain and helicase (Figure 1) [5,18,19].

The virological information obtained to date concerning ORF4 of HEV-1 is extremely limited, and a study published in 2016 indicated that the ORF4 product of HEV-1 interacts with multiple viral proteins to form a viral replication complex containing RdRp, Hel, and X proteins, and the ORF4-related protein promotes RdRp activity by interacting with host eEF1α1 and tubulin β, which is indispensable for the replication of HEV-1 [19]. However, there has been no report to confirm the role of ORF4 during the natural course of HEV-1 replication and infection. In the present study, we produced ORF4-deficient HEV-1s by a reverse genetic system to investigate whether ORF4 affects the replication and infectivity of HEV-1.

## 2. Materials and Methods

### 2.1. Design of ORF4-Defective HEV-1 and the In Vitro Transcription of HEV-1 RNA

An entire genome cDNA of HEV-1 was synthesized based on the nucleotide sequence derived from a subtype 1a strain (GenBank accession no. LC061267). The putative ORF4 of HEV-1 (nucleotides [nt] 2836–3255) encodes 139 amino acids (aa). The start ATG codon was changed to TTG (A2836T) or ACG (T2837C). Because we observed a downstream in-frame ATG codon (nt 2884–2886) near the N-terminal of ORF4, we also designed a mutation, T2885C. The mutations T2837C and T2885C did not result in the ORF1 aa changes, but A2836T resulted in an aa mutation, D937V (Asp to Val) in ORF1 and T2885C resulted in a aa mutation (Met to Thr) in ORF4 (Figure 1, Table 1).

The complete RNA transcripts of the wild-type HEV-1 and four mutant viruses were synthesized, and we added a T7 RNA polymerase promoter sequence to them at the 5′ end and an *Xba*I-site at the 3′ end. These five constructs were cloned into pUC57 vector to generate plasmids pUC57-HEV-1, pUC57-A2836T, pUC57-T2837C, pUC57-T2885C, and pUC57-T2837C/T2885C, respectively (GeneScript, Piscataway, NJ, USA) (Table 1). All plasmids were linearized with *Xba*I and purified by phenol/chloroform extraction. Capped HEV RNA was synthesized using an mMESSAGE mMACHINE T7 transcription kit (Ambion, Austin, TX, USA) according to the manufacturer’s recommendations.

The synthesized capped RNAs were treated with TURBO DNase and purified by lithium chloride precipitation and named HEV-1^RNA^, A2836T^RNA^, T2837C^RNA^, T2885C^RNA^, and T2837C/T2885C^RNA^, respectively (Table 1). HEV-1^RNA^ encoded the wild-type HEV-1 genome sequence. A2836T^RNA^ lost the ORF4 and gained an aa mutation of D937V in the ORF1. T2837C^RNA^ lost the ORF4 without ORF1 aa mutation. T2885C^RNA^ kept the ORF4 with an aa mutation (M17T) in the ORF4. T2837C/T2885C^RNA^ lost the ORF4 and obtained an aa mutation (M17T) in the ORF4. Capped RNA sequences were confirmed by a next-generation sequencing analysis before transfection.

### 2.2. Cell Culture and Transfection

A human hepatocarcinoma cell line, PLC/PRF/5 (JCRB0406), was grown in Dulbecco’s Modified Eagle’s Medium (DMEM) supplemented with 10% (*v*/*v*) heat-inactivated fetal bovine serum (FBS; Nichirei Biosciences, Tokyo), 100 U/mL penicillin, and 100 µg/mL streptomycin (Gibco, Grand Island, NY, USA) at 37 °C in a humidified 5% CO_2_ atmosphere and passaged every 3 days.

For transfection, 5 × 10^5^ PLC/PRF/5 cells were cultured in a 25 cm^2^ tissue culture flask for 24 h and then washed with phosphate-buffered saline (PBS) and supplemented with 6.6 mL of the new medium with 10% FBS. The transfection was performed using a *Trans*IT-mRNA Transfection Kit (Mirus Bio, Madison, WI, USA). Briefly, 6.6 µg of the capped parental or mutated virus RNAs were combined with 630 µL of Opti-MEM (Gibco), and then 13.2 µL of mRNA Boost Reagent and 13.2 µL of *Trans*IT-mRNA reagent were added to the mixture. After 5 min of incubation at room temperature, the mixture was added to the PLC/PRF/5 cells containing 6.6 mL of medium. After a 12 h incubation at 37 °C, the cells were washed three times with PBS and the medium was replaced with 10 mL of maintenance medium: medium 199 (Invitrogen, Carlsbad, CA, USA) containing 2% (*v*/*v*) heat-inactivated FBS and 10 mM MgCl_2_. Further incubation was performed at 36 °C. The medium was replaced with the new medium every 4 days, and the culture supernatant was used for the detection of HEV-1 RNA.

### 2.3. Infection of Mongolian Gerbils and the Sample Collection

Fifteen 6-week-old female Mongolian gerbils (MON/Jms/GbsSlc, SLC, Hamamatsu, Japan) were used. All were individually housed in a Biosafety Level-2 facility and were tested and confirmed to be negative for both the serum anti-HEV IgG antibodies and the HEV RNA. The gerbils were randomly separated into five groups (n = 3 each). Each gerbil was intraperitoneally inoculated with 1 mL of HEV-1p0, A2836Tp0, T2837Cp0, T2885Cp0, or T2837C/T2885Cp0 containing 10^8^ copies/mL of the viral RNA.

Fecal specimens were collected every 3 or 4 days, and 10% stool suspensions were used for the detection of the viral RNA. Serum samples were collected only at the end of the experiment and were used for the detection of the virus RNA, anti-HEV IgG antibodies, and ALT. At the end of the experiment, the gerbils were euthanized by exsanguination from the heart under anesthesia.

The animal experiments were reviewed and approved by the institutional ethics committee of our institution and were performed according to the Guides for Animal Experiments issued by Japan’s National Institute of Infectious Diseases under code 121025 (27 May 2021).

### 2.4. Detection of HEV-1 RNA

The extraction of viral RNA from 200 µL of the cell culture supernatants and from the 10% stool suspensions was carried out by a MagNA Pure 96 System (Roche Applied Science, Mannheim, Germany) with a MagNA Pure 96 DNA and Viral NA Small Volume Kit (Roche Applied Science). HEV-1 RNA was examined by a one-step real-time reverse transcription-quantitative polymerase chain reaction (RT-qPCR) using TaqMan Fast Virus 1-step Master Mix (Applied Biosystems, Foster City, CA, USA) and a QuantStudio 3 Real-Time PCR System (Applied Biosystems). The RT-qPCR was carried out under the condition of 5 min at 50 °C, 20 s incubation at 95 °C, followed by 40 cycles for 3 s at 95 °C and 30 s at 60 °C with a forward primer, JVHEVF (5′-GGTGGTTTCTGGGGTGAC-3′ nt 5346–5363), a reverse primer, JVHEVR (5′-AGGGGTTGGTTGGATGAA-3′ nt 5393–5415), and a probe, JVHEVP (5′-FAM-TGATTCTCAGCCCTTCGC-TAMRA-3′ nt 5369–5386) [21]. A 10-fold serial dilution of the capped HEV-1 RNA (10^7^ to 10^1^ copies) was used as the standard for the quantitation of the viral genome copy numbers. Amplification data were collected and analyzed with QuantStudio Design & Analysis software ver. 1.5 (Applied Biosystems).

A semi-nested reverse transcription-polymerase chain reaction (RT-PCR) was performed to amplify 507 base pairs (bp) of the ORF1 genome (nt 2794–3300) that covered the entire genome of ORF4. Five microliters of the cDNA was used for the first PCR in 50 µL of the reaction mixture containing an external forward primer, ORF4F1 (5′-TATACCAGGTGCCAATCGGT-3′ nt 2695–2714) and a reverse primer, ORF4R3 (5′-ACCACGGATCAACTCGCATA-3′ nt 3301–3200). Two microliters of the first PCR product was used for the nested PCR with an inner forward primer, ORF4F2 (5′-ACTTGCTGCCAGATGGTTTG-3′ nt 2774–2793) and ORF4R3. The PCR amplification was performed under the following conditions: inoculation at 94 °C for 60 s, followed by 35 cycles of 30 s at 94 °C, 30 s at 55 °C, and 75 s at 72 °C, and a final extension at 72 °C for 7 min. The PCR products were purified using a QIAquick PCR purification kit (Qiagen, Hilden, Germany), and the nucleotide sequencing was carried out with primers ORF4F2 and ORF4R3 using an ABI 3130 Genetic Analyzer Automated Sequencer (Applied Biosystems) and a BigDye Terminator Cycle Sequencing Ready Reaction kit (Applied Biosystems) according to the manufacturer’s instructions. The sequence analysis was performed using the Genetyx ver. 11.0.4 software program (Genetyx, Tokyo).

### 2.5. Detection of Anti-HEV IgG Antibodies

Anti-HEV IgG antibodies were detected by an enzyme-linked immunosorbent assay (ELISA) using virus-like particles (VLPs) of HEV-1 as the antigen as described [22]. Briefly, flat-bottomed 96-well polystyrene microplates (Immulon 2, Dynex Technologies, Chantilly, VA, USA) were coated with 100 ng/well of the VLPs, and the duplicates of the 1:200-diluted serum samples were used. Horseradish peroxidase (HRP)-conjugated rabbit anti-Mongolian gerbil IgG antibody (H+L) (1:1000) (Bioss, Boston, MA, USA) or HRP-conjugated goat anti-monkey IgG-heavy and light-chain antibody (Bethyl Laboratories, Montgomery, TX, USA) were used as the secondary antibody. The cut-off values for the detection of Mongolian gerbil and monkey anti-HEV IgG were 0.150 and 0.182, respectively [23,24].

### 2.6. Liver Enzyme Level

Alanine aminotransferase (ALT) values in the gerbil sera were monitored using a Fuji Dri-Chem Slide GPT/ALT-PIII kit (Fujifilm, Saitama, Japan) [23].

### 2.7. Expression of ORF4-Related Protein

The ORF4 genome containing the *Bam*H I site before the start codon and the *Not* I site after the stop codon was amplified by PCR with the primers ORF4*Bam*H I (5′-GGATCCATGTTGCACGGACAGCGAAT-3′) and ORF4*Not* I (5′-GCGGCCGCCTAAGTCGGGCCTGATGGCG-3′). The amplified DNA fragments were purified with a Qiagen Gel purification kit (Qiagen, Valencia, CA, USA) and cloned into a TA 2.1 cloning vector (Invitrogen, San Diego, CA, USA), then ligated with an expression-vector pET32a (+) to generate pET32a-ORF4 and used to transform an *E. coli* strain BL21 (DE3). The *E. coli* was incubated in Luria–Bertani medium containing 100 μg/mL ampicillin at 37 °C until the absorbance at 600 nm reached 0.6. Then, 0.1 mM isopropyl beta-d-thiogalactopyranoside (IPTG) was added to the culture and incubated for 3 h.

### 2.8. Western Blot Analysis

The viral proteins were detected by Western blot analysis (WB). The virus-infected PLC/PRF/5 cells or pET32a-ORF4-transformed BL21 (DE3) were separated by centrifugation at 10,000× *g* for 5 min and suspended with PBS. The viral proteins were separated by 5–20% sodium dodecyl sulfate-polyacrylamide gel electrophoresis (SDS-PAGE) and electrophoretically transferred onto a nitrocellulose membrane. The membrane was then blocked with 5% skim milk in 50 mM Tris-HCl (pH 7.4) containing 150 mM NaCl, and incubated with cynomolgus monkey anti-HEV-1 serum (1:500 dilution) with PBS-T containing 1% skim milk [25]. Detection of the monkey IgG antibody was achieved using alkaline phosphatase-conjugated rabbit anti-monkey IgG (whole molecular) (1:1000 dilution) (Sigma-Aldrich, St. Louis, MO, USA). Nitroblue tetrazolium chloride and 5-bromo-4-chloro-3-indolyl phosphate P-toluidine were used as the coloring agents (Bio-Rad Laboratories, Hercules, CA, USA).

## 3. Results

### 3.1. Generation of Infectious HEV-1 and ORF4-Defective HEV-1s

Capped RNAs encoding an entire genome derived from the wild-type HEV-1 and those derived from the ORF4-defective genomes were prepared by in vitro transcription as described above. The capped viral RNAs, i.e., HEV-1^RNA^, A2836T ^RNA^, T2837C^RNA^, T2885C^RNA^, and T2837C/T2885C^RNA^, were used to transfect PLC/PRF/5 cells. The transfections were performed using triplicate samples.

The virus RNA was detected in the HEV-1^RNA^-transfected cell culture supernatant on day 4 post-transfection (p.t.), at 3.0 × 10^7^ copies/mL. The copy numbers temporarily decreased to 2.0 × 10^7^ copies/mL on day 8 p.t. and then increased again and reached 1.8 × 10^8^ copies/mL on day 24 p.t.; the copy numbers were then maintained at ~3.6 × 10^8^ to 7.3 × 10^8^ copies/mL until day 56 p.t. Similar replication patterns were observed in the T2837C^RNA^, T2885C^RNA^, and T2837C/T2885C^RNA^-transfected cells.

**Figure 2:**
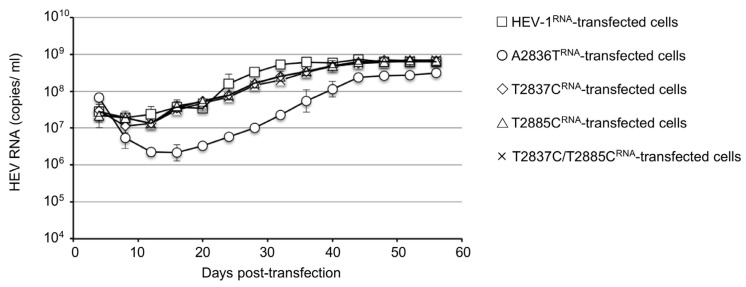
Generation and replication of the original and ORF4-defective HEV-1 in PLC/PRF/5 cells. PLC/PRF/5 cells were transfected with capped HEV-1^RNA^, A2836T^RNA^, T2837C^RNA^, T2885C^RNA^, and T2837^RNA^/T2885^RNA^, respectively. The culture supernatant was collected every 4 days, and the viral RNA was measured by RT-qPCR. The viral RNA copy numbers are shown in the HEV-1^RNA^-transfected cells (≤), A2836T^RNA^-transfected cells (○), T2837C^RNA^-transfected cells (◊), T2885C^RNA^-transfected cells (△), and T2837C/T2885C^RNA^-transfected cells (×).

In contrast, the virus RNA was detected in the A2836T^RNA^-transfected cell culture supernatant on day 4 p.t. at 3.3 × 10^7^ copies/mL, and the copy numbers decreased to 2.2 × 10^6^ copies/mL on day 12 p.t. and then gradually increased. Although the viral copy numbers increased to 1.3 × 10^8^ copies/mL on day 40 p.t. and were maintained at ~2.3 × 10^8^ to 3.2 × 10^8^ copies/mL until day 56 p.t., the RNA level was lower than those detected in the HEV-1^RNA^, T2837C^RNA^, T2885C^RNA^, and T2837C/T2885C^RNA^-transfected cells (Figure 2). These results indicated that the mutation of M1T and M17T of ORF4 did not affect the HEV-1 replication in the PLC/PRF/5 cells. In contrast, the mutation of D937V in the ORF1 reduced unequivocally the replication. We designated the viruses recovered from the supernatants as HEV-1p0, A2836Tp0, T2837Cp0, T2885Cp0, and T2837C/T2885Cp0, respectively.

For a confirmation of the infectivity of HEV-1p0, A2836Tp0, T2837Cp0, T2885Cp0, and T2837C/T2885Cp0, the culture supernatant was collected on day 52 p.t., and the virus RNA titer was adjusted to 1 × 10^8^ copies/mL and used to inoculate PLC/PRF/5 cells. Triplicate samples were used for inoculation. The virus RNA titers on day 4 post-infection (p.i.) in the HEV-1p0-, T2837Cp0-, T2885Cp0-, and T2837C/T2885Cp0-inoculated cell culture supernatants were 1.37 × 10^6^, 2.80 × 10^6^, 2.61 × 10^6^, and 2.63 × 10^6^ copies/mL, respectively. These viral RNAs then increased in a similar pattern and reached >10^8^ copies/mL on day 24 p.i.; by day 40 p.i., the viral RNAs reached peaks at 7.22 × 10^8^ to 7.56 × 10^8^ copies/mL in the HEV-1p0-, T2837Cp0-, T2885Cp0- and T2837C/T2885Cp0-inoculated cells.

**Figure 3:**
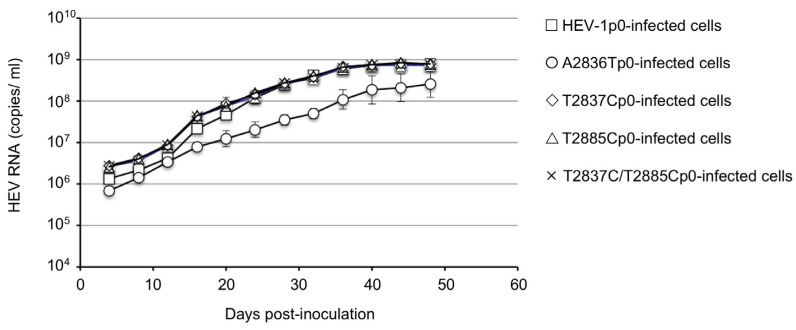
Infectivity of the original and ORF4-defective HEV-1s in PLC/PRF/5 cells. PLC/PRF/5 cells were inoculated with HEV-1p0, A2836Tp0, T2837Cp0, T2885Cp0, and T2837C/T2885Cp0 containing the same RNA copy number, respectively. The culture supernatant was collected every 4 days, and the viral RNA was measured by RT-qPCR. The RNA titers are shown in the HEV-1p0-infected cells (≤), A2836Tp0-infected cells (○), T2837Cp0-infected cells (◊), T2885Cp0-infected cells (△), and T2837C/T2885Cp0-infected cells (×).

However, the RNA titers of the A2836Tp0-inoculated cells on days 4, 24, and 40 p.i. were 6.88 × 10^5^, 2.16 × 10^7^, and 2.21 × 10^8^ copies/mL, respectively, and these values are clearly lower than those detected in the cells infected with the other four viruses (Figure 3). These results further confirmed that two in-frame ATG mutations in ORF4 did not affect the infectivity in PLC/PRF/5 cells, but an accompanying mutation, D937V, in the X-domain of ORF1 reduced the virus replication in PLC/PRF/5 cells. The viruses recovered from the supernatants of p0 virus-infected cells were designated HEV-1p1, A2836Tp1, T2837Cp1, T2885Cp1, and T2837C/T2885Cp1, respectively.

### 3.2. Nucleotide Sequence Analyses of ORF4-Defective HEV-1s

To confirm whether the mutated sequences were stable during the virus replication, we used the HEV-1p0, A2836Tp0, T2837Cp0, T2885Cp0, and T2837C/T2885Cp0 collected on day 60 p.t. and HEV-1p1, A2836Tp1, T2837Cp1, T2885Cp1, and T2837C/T2885Cp1 collected on day 44 p.i. for the amplification of 507 bp that covered the entire ORF4 genome (nt 2794–3300) by RT-PCR. The nucleotide sequences from all 30 samples (three samples each from the 10 supernatants) were identical to each respective nucleotide sequence. These results indicated that no mutations occurred in ORF4 and the mutations of A2836T, T2837C, and T2885C were genetically stable during the virus replication in the PLC/PRF/5 cells.

### 3.3. Infectivity of the ORF4-Defective HEV1s In Vivo

**Figure 4:**
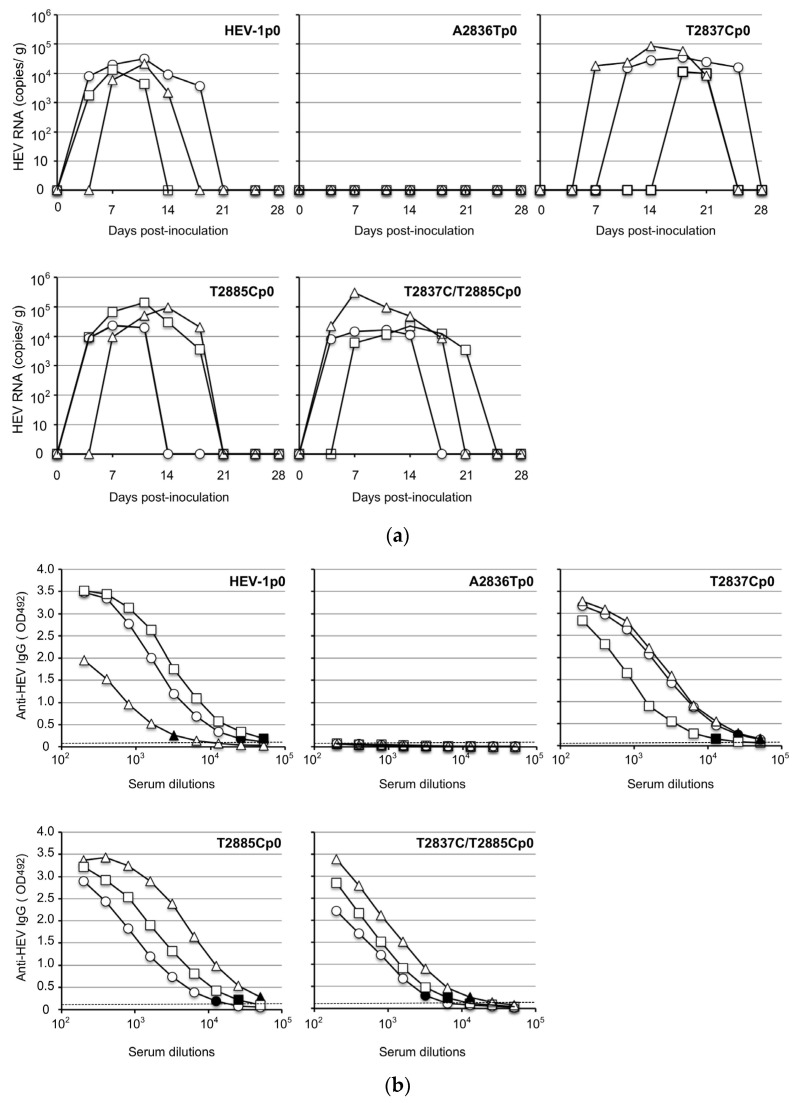
Infection of original and ORF4-defective viruses in Mongolian gerbils. Fifteen Mongolia gerbils were randomly separated into five groups (n = 3 per group). Individual gerbils are indicated by ○, △, and ☐. Each group received HEV-1p0, A2836Tp0, T2837Cp0, T2885Cp0, or T2837C/T2885Cp0 via intraperitoneal injection. The kinetics of the viral RNA in the fecal specimens were measured by RT-qPCR (**a**). The serum samples were collected at the end of the experiment (day 28 post-inoculation [p.i.]), and the anti-HEV-IgG antibody titers were determined by an ELISA with the virus-like particles (VLPs) of HEV-1 as the antigens (**b**). *Dotted lines*: the cut-off values. The minimum endpoints of the antibody titers are *blackened*.

For the investigation of the infectivity of the ORF4-defective viruses in vivo, HEV-1p0, A2836Tp0, T2837Cp0, T2885Cp0, and T2837C/T2885Cp0 containing 1.0 × 10^8^ copies/mL of the virus RNA were intraperitoneally inoculated into Mongolian gerbils as described in the Materials and Methods. The virus RNA in the fecal specimens was monitored by RT-qPCR. As shown in Figure 4a, the virus RNAs were detected in all of the fecal specimens from the HEV-1p0-, T2837Cp0-, T2885Cp0-, and T2837C/T2885Cp0-inoculated gerbils, although the detectable periods differed. The virus RNAs reached peaks around day 14 p.i. and the titers ranged from 1.4 × 10^4^ to 3.2 × 10^4^ copies/g in the HEV-1p0-inoculated gerbils, from 1.1 × 10^4^ to 8.5 × 10^4^ copies/g in the T2837Cp0-inoculated gerbils, from 2.4 × 10^4^ to 1.4 × 10^5^ copies/g in the T2885Cp0-inoculated gerbils, and from 1.6 × 10^4^ to 9.3 × 10^4^ copies/g in the HEV- T2837C/T2885Cp0-inoculated gerbils. The virus RNA titers then decreased and became undetectable in all of the HEV-inoculated gerbils on day 28 p.i. In contrast, no virus RNA was detected in the three A2836Tp0-inoculated gerbils (Figure 4a).

All of the gerbils were euthanized on day 28 p.i., and serum samples were collected for the detection of the anti-HEV IgG antibodies and ALT. The anti-HEV IgG antibodies were detected in the serum of all of the animals inoculated with HEV-1p0, T2837Cp0, T2885Cp0, or T2837C/T2885Cp0, and the titers were 1:6400–51,200 in the HEV-1p0-inoculated gerbils, 1:6400–12,800 in the T2837Cp0-inoculated gerbils, 1:12,800–51,200 in the HEV-T2885Cp0-inoculated gerbils, and 1:3200–1:12,800 in the T2837C/T2885Cp0-inoculated gerbils. No antibody was detected in any of the three A2836Tp0-inoculated animals (Figure 4b).

The ALT values ranged from 41 IU/L to 86 IU/L in the gerbils on day 28 p.i., and there was no significant difference among the HEV-1p0-, T2836Ap0-, T2837Cp0-, T2885Cp0-, and T2837C/T2885Cp0-inoculated animals (Table 2).

To confirm whether infection occurred actually by ORF4-defective HEV-1, we amplified a portion of the virus RNA genome by RT-PCR using the fecal samples. The virus RNA-positive stool suspensions collected from T2837Cp0-, T2885Cp0-, and T2837C/T2885Cp0-infected gerbils were concentrated by ultracentrifugation and used for the virus RNA extraction; in addition, 507 bp of ORF1 containing entire genome of ORF4 was amplified by RT-PCR. The nucleotide sequence analyses confirmed that no mutations occurred in ORF4, and T2837Cp0 and T2885Cp0, which contain in-frame ATG mutations but no accompanying aa mutation in ORF1, were genetically stable during the replication in the gerbils, and that the putative ORF4 was not essential for HEV infection in vivo. In contrast, A2836Tp0 containing the D937V change in the X-domain of the ORF1 did not infect the gerbils, demonstrating that this mutation was critical for the HEV-1 infectivity.

### 3.4. Detection of Viral Protein in the Wild-type and the ORF4-defective HEV-1s-Infected PLC/PRF/5 Cells

**Figure 5:**
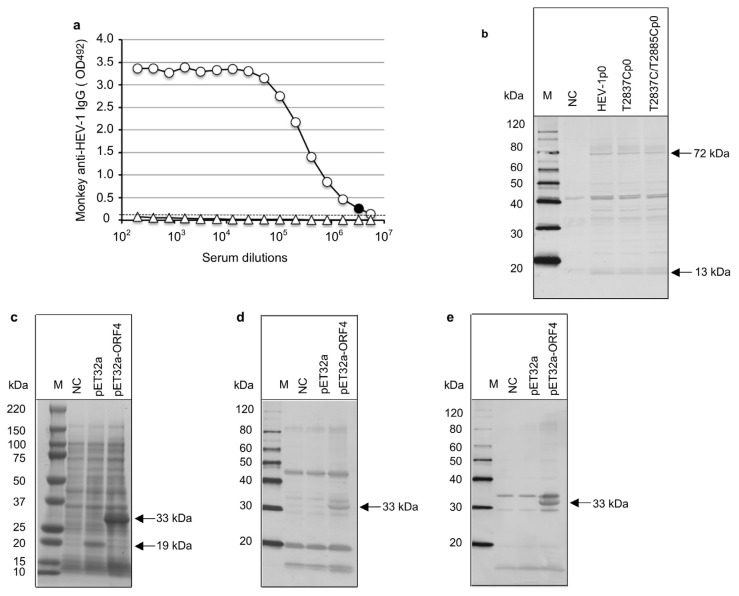
Detection of viral proteins in HEV-1-infected PLC/PRF/5 cells. A cynomolgus monkey’s serum bearing the anti-HEV-1 IgG antibody titer 1:3,276,800 by ELISA was used for Western blot analyses. The minimum endpoints of the antibody titers are *blackened* (**a**). HEV-1p0-, T2837Cp0-, and T2837C/T2885Cp0-infected PLC/PRF/5 cells were harvested on day 48 p.i., and the virus proteins were detected by Western blot analysis (**b**). ORF4 was expressed by an *E. coli* expression system, and the related protein was analyzed by SDS-PAGE (**c**) and a Western blot analysis with monkey anti-HEV-1 serum (**d**) and anti-HEV-7 serum (**e**). M: molecular weight, NC: no-infected PLC/PRF/5 cells (**b**) or no-transformed BL21 (DE3) cells (**c**–**e**). pET32a (+): vector pET32a (+)-transformed BL21 (DE3). pET32aORF4: pET32aORF4-transformed BL21 (DE3).

For the investigation of whether the ORF4-encoded protein appeared in the virus-infected cells, we collected HEV-1p0-, HEV-T2837Cp0-, and HEV-T2837C/T2885Cp0-infected PLC/PRF/5 cells on day 48 p.i., and the viral proteins were analyzed by WB with monkey anti-HEV-1 serum collected from an HEV-1 (LC061267)-infected cynomolgus monkey [25]. The antibody titer of anti-capsid protein was as high as 1:3,276,800 by ELISA (Figure 5a).

Two protein bands were detected: one ~72 kDa corresponding to the capsid protein and the other ~13 kDa corresponding to the ORF3 protein (Figure 5b). However, no other extra protein band was detected in the wild-type virus-infected cells compared to the two ORF4-defective HEV-1-infected cells, demonstrating no evidence of the putative viral protein(s) derived from ORF4 appeared in HEV-1-infected cells.

To confirm whether the antibody against ORF4 was induced in the HEV-1 infected monkeys, we cloned the ORF4 into the vector pET32a (+) and expressed the ORF4 in *E. coli* strain BL21 (DE3). Since ORF4 was inserted between the *Bam*H I and *Not* I in the MCS region of pET32a (+), the expressed fusion protein should have 306 aa (167 aa from the vector and 139 aa from ORF4) and the molecular weight was calculated as ~33 kDa. As shown in Figure 5c, an approx. 19 kDa protein (p19) derived from the 167 aa was detected in vector pET32a (+)-transformed cells, whereas an approx. 33 kDa protein (p33) was observed in the pET32a-ORF4-transformed cells (Figure 5b). The WB analyses showed that p33 reacted not only with monkey anti-HEV-1 serum (Figure 5d) but also with monkey anti-HEV-7 serum [26] (Figure 5e), suggesting that sera from the HEV-1-infected monkey had non-specific reactivity against p33.

## 4. Discussion

Although a putative ORF4 has been observed in the HEV-1 genome, its function has not been clear and the information regarding this ORF is limited. A reverse genetic system, which is a powerful tool to produce infectious HEVs from cloned cDNAs, allowed us to explore the function of ORF4 in the present study. We directly mutated the putative initiator methionine in the ORF4, ATG to TTG (M to L) or ATG to ACG (M to T), in order to disable the translation from the ORF4. The results demonstrated that these mutant viruses retained the ability to replicate in PLC/PRF/5 cells as the wild-type HEV-1 did, although a later-developed mutant virus with an accompanying aa mutation (D937V) in ORF1 exhibited slower virus replication, demonstrating that the ORF4 was not essential for the HEV-1 replication in PLC/PRF/5 cells. In addition, the mutation in the putative second start cordon (nt 2854–2856) alone and simultaneous mutations with the first start codon (nt 2836–2838) did not affect the replication or infection in vitro, demonstrating that this putative smaller ORF4 is also not essential for HEV-1 replication and infection in vitro.

Cynomolgus and rhesus monkeys are known to be susceptible to HEV infection, and they have provided a good animal model for examinations of the infectivity of HEV-1 [25,27,28,29]. However, the fact that most of these monkeys have been exposed to HEV made us hesitant to use them for the infectious experiments [24,30]. Fortunately, our previous findings indicated that Mongolian gerbils are susceptible to HEV-1 [23]. Our present findings revealed that the ORF4-defective HEV-1 had an ability to infect Mongolian gerbils unless the mutations in the overlapping ORF1 were accompanied by the non-synonymous change, suggesting that aa D937 in the ORF1 is important for HEV-1 infection.

Although it was reported that anti-ORF4 antibodies were detected in the serum of an HEV-1-infected patient [19], no corresponding protein(s) derived from ORF4 were confirmed in the HEV-1-infected cells. In the present study, no ORF4-related protein was detectable by WB even when a 1:3,267,800 titer of the monkey anti-HEV-1 IgG antibody was used. No evidence indicating that the viral protein(s) related to the ORF4 were expressed in the HEV-1-infected PLC/PRF/5 cells was obtained.

Our results demonstrated that ORF4 is not essential for HEV-1 replication and infectivity, in contradiction to another report [19]. In different HEV-1 isolates, ORF4 had the potential to encode 90aa (LC225387), 139 aa (LC061267), 147aa (NC_001434), and 158aa (AF444002), and the N-terminal 124 aa of ORF4 was thought to be sufficient for interacting with other viral and host proteins [19]. However, the ORF4 of the subtype 1g (LC225387, MH504161, LC314156) encodes 90 aa, and whether this shorter protein is sufficient to interact with other viral and host proteins is unclear. Further studies are required to clarify the role of ORF4 in the life cycles of HEV-1.

## Figures and Tables

**Table 1:** Nucleotide mutations and resulted amino acid mutations in each construct of HEV-1.

Plasmid	RNA	Nucleotide Position	ORF Positions	Nucleotide Mutations	Amino Acid Positions	Amino Acid Changes
pUC57HEV-1	HEV-1^RNA^					
pUC57A2836T	A2836T^RNA^	2836	ORF4: 1	ORF4: A to T (Atg to Ttg)	ORF4: 1	ORF4: Met to Leu
			ORF1: 2813	ORF1: A to T (gAt to gTt)	ORF1: 937	ORF1: Asp to Val
pUC57T2837C	T2837C^RNA^	2837	ORF4: 2	ORF4: T to C (aTg to aCg)	ORF4: 1	ORF4: Met to Thr
			ORF1: 2814	ORF1: T to C (gaT to gaC)	ORF1: 937	ORF1: Non
pUC57T2885C	T2885C^RNA^	2885	ORF4: 50	ORF4: T to C (aTg to aCg)	ORF4: 17	ORF4: Met to Thr
			ORF1: 2862	ORF1: T to C (gaT to gaC)	ORF1: 953	ORF1: Non
pUC57T2837C/	T2837C/ T2885C^RNA^		ORF4: 2	ORF4: T to C (aTg to aCg)	ORF4: 1	ORF4: Met to Thr
		2837	ORF1: 2814	ORF1: T to C (gaT to gaC)	ORF1: 937	ORF1: Non
T2885C		2885	ORF4: 50	ORF4: T to C (aTg to aCg)	ORF4: 17	ORF4: Met to Thr
			ORF1: 2862	ORF1: T to C (gaT to gaC)	ORF1: 953	ORF1: Non

**Table 2:** ALT (IU/L) in the sera collected from HEV-1-inoculated Mongolian gerbils on day 28 p.i.

HEV Strain	Gerbil 1	Gerbil 2	Gerbil 3
HEV-1	55	49	52
T2837C	57	86	60
A2836T	49	50	54
T2885C	42	56	61
T2837C/ T2885C	41	47	53

## Data Availability

The sequences of HEV used in this study have been assigned (GenBank accession no. LC061267).
